# Association study of polymorphisms in the neutral amino acid transporter genes *SLC1A4*, *SLC1A5 *and the glycine transporter genes *SLC6A5*, *SLC6A9 *with schizophrenia

**DOI:** 10.1186/1471-244X-8-58

**Published:** 2008-07-18

**Authors:** Xiangdong Deng, Noriaki Sagata, Naoko Takeuchi, Masami Tanaka, Hideaki Ninomiya, Nakao Iwata, Norio Ozaki, Hiroki Shibata, Yasuyuki Fukumaki

**Affiliations:** 1 Division of Human Molecular Genetics, Research Center for Genetic Information, Medical Institute of Bioregulation, Kyushu University, Fukuoka 812-8582, Japan; 2 Fukuoka Prefectural Dazaifu Hospital Psychiatric Center, Dazaifu, Fukuoka, Japan; 3 Department of Psychiatry, Fujita Health University School of Medicine, Toyoake, Aichi 470-1192, Japan; 4 Department of Psychiatry, Graduate School of Medicine, Nagoya University, Nagoya, Japan

## Abstract

**Background:**

Based on the glutamatergic dysfunction hypothesis for schizophrenia pathogenesis, we have been performing systematic association studies of schizophrenia with the genes involved in glutametergic transmission. We report here association studies of schizophrenia with *SLC1A4*, *SLC1A5 *encoding neutral amino acid transporters ASCT1, ASCT2, and *SLC6A5*, *SLC6A9 *encoding glycine transporters GLYT2, GLYT1, respectively.

**Methods:**

We initially tested the association of 21 single nucleotide polymorphisms (SNPs) distributed in the four gene regions with schizophrenia using 100 Japanese cases-control pairs and examined allele, genotype and haplotype association with schizophrenia. The observed nominal significance were examined in the full-size samples (400 cases and 420 controls).

**Results:**

We observed nominally significant single-marker associations with schizophrenia in SNP2 (*P *= 0.021) and SNP3 (*P *= 0.029) of *SLC1A4*, SNP1 (*P *= 0.009) and SNP2 (*P *= 0.022) of *SLC6A5*. We also observed nominally significant haplotype associations with schizophrenia in the combinations of SNP2-SNP7 (*P *= 0.037) of *SLC1A4 *and SNP1-SNP4 (*P *= 0.043) of *SLC6A5*. We examined all of the nominal significance in the Full-size Sample Set, except one haplotype with insufficient LD. The significant association of SNP1 of *SLC6A5 *with schizophrenia was confirmed in the Full-size Sample Set (*P *= 0.018).

**Conclusion:**

We concluded that at least one susceptibility locus for schizophrenia may be located within or nearby *SLC6A5*, whereas *SLC1A4*, *SLC1A5 *and *SLC6A9 *are unlikely to be major susceptibility genes for schizophrenia in the Japanese population.

## Background

Schizophrenia is a devastating mental disorder that affects about 1% of worldwide populations [1], and genetic factors are known to play a crucial role in its pathogenesis [2]. The successful treatment with dopamine antagonists on the positive symptomatology of the disease suggests a crucial role of dopamine in the pathophysiology of schizophrenia. However, due to the poor effects of dopamine antagonists against the negative and cognitive symptoms of schizophrenia, other neurotransmitter systems than dopamine, such as glutamate are suggested to be involved in the pathogenesis of schizophrenia. Based on the fact that phencyclidine (PCP), the antagonist of N-methyl-D-aspartate (NMDA) glutamate receptor, induces schizophreniform psychosis, a glutamatergic dysfunction hypothesis has been proposed for the pathogenesis of schizophrenia [3-5]. This hypothesis has been supported by recent multiple reports of significant association of schizophrenia with glutamate receptor genes and with the genes related to glutamatergic transmission [Review, [6,7]]. The dopamine and glutamate hypothesis of schizophrenia are not independent, and in fact, glutamate-dopamine interaction has been supported by many preclinical and clinical findings [Review, [8]].

Other synaptic elements related to glutamate, such as transporters, also potentially affect glutamatergic neurotransmission. Excitatory amino acid transporters (EAATs) maintain extracellular glutamate concentrations within physiological levels by reuptaking synaptically released glutamate. Abnormalities of mRNA expression of EAATs were reported in the thalamus, prefrontal cortex, parahippocampal gyrus and striatum in schizophrenia [9-12]. Recently, we have reported the positive association of *SLC1A2 *and *SLC1A6*, the genes encoding EAAT2 and EAAT4, respectively with schizophrenia [13,14], providing support for the potential important roles of EAATs in schizophrenia.

Neutral amino acid transporters (ASCTs), which transport neutral amino acid (alanine, serine, cysteine and threonin) were identified based on nucleotide sequence homology to the EAATs [15,16]. The amino acid identity between EAATs and ASCTs is 40–44%. The functions of ASCTs in glutamate transmission have also been reported. ASCT1 not only mediates the efflux of glutamate from the neuron into the synaptic junction via Calcium-independent release, but also mediates the efflux of L-serine from glial cells and its uptake by neurons [17-19]. L-serine is used for syntheses of various biomolecules, including the co-agonists at NMDA glutamate receptor, D-serine and glycine. ASCT2 appears to play an important role in the glutamine-glutamate cycle between neurons and glia by facilitation the efflux of glutamine from glial cells [20]. Recently, Weis et al. reported significant decrease in ASCT1 immunoreactivity in the cingulate cortex, white matter, and striking loss of ASCT1 immunoreactivity in the hippocampus in schizophrenia. [21].

Glycine acts as an obligatory co-agonist at NMDA glutamate receptor to promote NMDA receptor function. In the central system, the actions of glycine are terminated by its rapid uptake into the nerve terminal and adjacent glial cells via high-affinity glycine transporters (GLYTs) [22]. Therefore, increasing synaptic level of glycine by inhibiton of its uptake could lead to enhance the activation of NMDA receptor. Both preclinical and clinical evidence have provided support for the utility of this modulatory approach, as well as the potential therapeutic value of GLYT1 inhibitors in the treatment of schizophrenia [Review, [23]]. Therefore the ASCTs and GLYTs genes are strong candidates for schizophrenia, as well as glutamate receptor and glutamate transporter genes.

In this study we report association studies of schizophrenia with total 21 SNPs distributed in genes *SLC1A4*, *SLC1A5*, *SLC6A5 *and *SLC6A9 *that encoding the neutral amino acid transporters ASCT1, ASCT2 and the glycine transporters GLYT2, GLYT1, respectively. SNPs were selected to cover the entire gene regions by linkage disequilibria (LD). To enhance the detection power of the study, we also examined the haplotype associations with the disease.

## Methods

### Human subjects

Blood samples were obtained from unrelated Japanese individuals who had provided written informed consent. We used 400 cases (mean age 47.2; 44.8% female) recruited from hospitals in Kyushu and Aichi areas and 420 unrelated controls (mean age 43.6; 44.0% female) recruited from the Kyushu and Aichi areas. We initially tested the association of the genes with schizophrenia using the Screening Sample Set: 100 out of 400 cases (mean age 49.5; 44.0% female) and 100 out of 420 controls (mean age 51.2; 44.0% female) recruited from the Kyushu area. All patients were diagnosed by the Diagnostic and Statistical Manual of Mental Disorders (DSM)-IV criteria [24]. The patients are all consecutive inpatients. The schizophrenia diagnoses were confirmed by several psychiatrists. We used another 16 healthy Japanese samples to test the frequencies of the candidate SNPs selected from the database. This study was approved by the Ethics Committee of Kyushu University, Faculty of Medicine. DNA samples were extracted from leukocytes by standard methods [25].

### SNP selection

We retrieved the primary SNP information from the dbSNP database [26]. Assuming the same size of the half length of LD (60 kb) as reported in Caucasians [27], we initially intended to select common SNPs every 30 kb in the three genes including all of the exonic SNPs. We tested the frequencies of the candidate SNPs, in the 16 healthy Japanese samples by the direct sequencing method. Out of them, common SNPs with minor allele frequencies over 10% were selected for further analyses. The SNPs in which significant deviation from Hardy-Weinberg equilibrium (HWE) observed in the 100 control samples were replaced by another SNP nearby. Since LD gaps (*D *' < 0.3) were observed in the initial SNP set after the LD analyses described below, we selected additional SNPs to fill the LD gaps.

**Figure 1:**
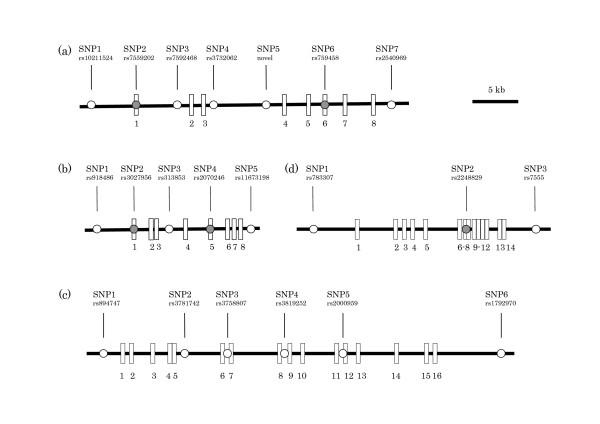
**Genomic organizations of each gene and locations of the SNPs.** (a) *SLC1A4 *spans over 34 kb and is composed of 8 exons. (b) *SLC1A5 *spans over 14 kb and is composed of 8 exons. (c) *SLC6A5 *spans over 55.6 kb and is composed of 16 exons. (d) *SLC6A9 *spans over 19.8 kb and is composed of 14 exons. Exons are shown as vertical bars with exon numbers. SNPs we analyzed are indicated by circles. Exonic SNPs are indicated by filled circles.

We finally selected the following 21 common SNPs distributed across the gene regions for further analyses: 7 of *SLC1A4*, SNP1, rs10211524; SNP2, rs7559202; SNP3, rs7592468; SNP4, rs3732062; SNP5, novel SNP in intron 3 (located on 33bp 5' to SNP rs7583682); SNP6, rs759458; SNP7, rs2540969, 5 of *SLC1A5*, SNP1, rs918486; SNP2, rs3027956; SNP3, rs313853; SNP4, rs2070246; SNP5, rs11673198, 6 of *SLC6A5*, SNP1, rs894747; SNP2, rs3781742; SNP3, rs3758807; SNP4, rs3819252; SNP5, rs2000959, SNP6, rs1792970 and 3 of *SLC6A9*, SNP1, rs783307; SNP2, rs2248829; SNP3, rs7555. The locations of the 21 SNPs are shown in Figure 1.

### Genotyping

The 21 SNPs were amplified as individual fragments by PCR as previously described [14]. The nucleotide sequences of each primer, PCR conditions and genotyping methods for each SNP are shown in Additional File 1. Because of the GC rich sequences of *SLC1A5 *region, we used Fail Safe PCR system (Epicentre Technologies) to optimize the PCR conditions when amplified SNP1, SNP2 and SNP5 of *SLC1A5*. We genotyped samples for SNP5 of *SLC6A5 *by polymerase chain reaction/restriction fragment length polymorphism (PCR-RFLP) and the other 20 SNPs by direct sequencing, as previously described [28]. The raw data of direct sequencing were compiled on PolyPhred [29] and/or Mutation Surveyor (SoftGenetics LLC).

### Statistical analyses

To control genotyping errors, Hardy-Weinberg equilibrium (HWE) for the genotype frequencies was checked in the control samples by the χ^2^-test (d.f. = 1). We evaluated the statistical differences in genotype and allele frequencies between cases and controls by Fisher's exact probability test. The magnitude of linkage disequilibrium (LD) was evaluated by caculating *D ' *using the haplotype frequencies estimated by the EH program, version 1.14 [30], and *D' *is represented graphically using the software Graphical Overview of Linkage Disequilibrium (GOLD) [31]. Statistical analysis of the haplotype association was carried out as previously described [32]. The significance level for all statistical tests was 0.05. We adjusted the *P *values of association studies for multiplicity using a false discovery rate (FDR) controlling procedure [33].

## Results

### Genotyping and SNP association analyses

We selected total 21 SNPs at an average interval of 7.8 kb for *SLC1A4*, 4.5 kb for *SLC1A5*, 14.2 kb for *SLC6A5 *and 18.9 kb for *SLC6A9 *to cover each entire gene region with LD. Since the average allele frequencies of the SNPs are 0.35, 0.30, 0.37 and 0.30 respectively, the expected detection powers for *SLC1A4*, *SLC1A5*, *SLC6A5 *and *SLC6A9 *are 0.84, 0.82, 0.84 and 0.82, respectively, under the multiplicative model with genotype relative risk = 1.8 [34]. Considering the high expected detection powers, we initially tested the single-marker association of the 21 SNPs with schizophrenia using the Screening Sample Set (100 cases and 100 controls) by the method described above, and investigated the association in the Full-size Sample Set (400 cases and 420 controls) only for the SNPs that showed significant single-marker or haplotype association with schizophrenia in the Screening Sample Set.

Table 1 shows the results of genotype and allele frequencies of SNPs in 100 case and 100 control samples. No significant deviation from HWE in control samples was observed in these SNPs (data not shown). We observed significant associations with schizophrenia in allele frequencies of SNP2 and SNP3 of *SLC1A4 *(*P *= 0.021, *P *= 0.029, respectively), and in genotype frequencies of SNP1 and SNP2 of *SLC6A5 *(*P *= 0.009, *P *= 0.022, respectively), although none of them survived after controlling the FDR at level 0.05 (n = 7 for *SLC1A4 *and n = 6 for *SLC6A5*).

**Table 1:** Genotype and allele frequencies of SNPs in each gene in the Screening Sample Set (100 cases and 100 controls)

Genes	Polymorphism	Genotype count			P*	Allele frequency (%)		P**
*SLC1A4*	SNP1	A/A	A/G	G/G		A	G	
	Schizophrenics	71	25	4	0.296	83.5	16.5	0.251
	Controls	61	35	4		78.5	21.5	
								
	SNP2	C/C	C/G	G/G		C	G	
	Schizophrenics	12	35	53	0.090	29.5	70.5	0.021
	Controls	6	27	67		19.5	80.5	
								
	SNP3	A/A	A/G	G/G		A	G	
	Schizophrenics	53	35	12	0.117	70.5	29.5	0.029
	Controls	67	26	7		80	20	
								
	SNP4	A/A	A/G	G/G		A	G	
	Schizophrenics	25	45	30	0.315	47.5	52.5	0.547
	Controls	17	54	29		44	56	
								
	SNP5	C/C	C/T	T/T		C	T	
	Schizophrenics	64	33	3	0.778	80.5	19.5	0.605
	Controls	68	30	2		83	17	
								
	SNP6	A/A	A/G	G/G		A	G	
	Schizophrenics	1	22	77	0.096	12	88	0.123
	Controls	7	22	71		18	82	
								
	SNP7	C/C	C/T	T/T		C	T	
	Schizophrenics	23	42	35	0.194	44	56	0.316
	Controls	23	53	24		49.5	50.5	
								

*SLC1A5*	SNP1	A/A	A/G	G/G		A	G	
	Schizophrenics	63	26	11	0.172	76.0	24.0	0.813
	Controls	60	35	5		77.5	22.5	
								
	SNP2	C/C	C/G	G/G		C	G	
	Schizophrenics	36	45	19	0.964	58.5	41.5	0.839
	Controls	37	46	17		60.0	40.0	
								
	SNP3	C/C	C/T	T/T		C	T	
	Schizophrenics	11	34	55	0.816	28.0	72.0	> 0.999
	Controls	9	37	54		27.5	72.5	
								
	SNP4	C/C	C/T	T/T		C	T	
	Schizophrenics	51	40	9	0.819	71.0	29.0	> 0.999
	Controls	52	37	11		70.5	29.5	
								
	SNP5	C/C	C/T	T/T		C	T	
	Schizophrenics	51	41	8	0.775	71.5	28.5	1.000
	Controls	53	37	10		71.5	28.5	

*SLC6A5*	SNP1	A/A	A/G	G/G		A	G	
	Schizophrenics	17	65	18	0.009	49.5	50.5	0.109
	Controls	35	46	19		58.0	42.0	
								
	SNP2	C/C	C/T	T/T		C	T	
	Schizophrenics	20	64	16	0.022	52.0	48.0	0.107
	Controls	37	47	16		60.5	39.5	
								
	SNP3	C/C	C/T	T/T		C	T	
	Schizophrenics	50	40	10	0.757	70.0	30.0	0.666
	Controls	45	45	10		67.5	32.5	
								
	SNP4	A/A	A/G	G/G		A	G	
	Schizophrenics	6	40	54	0.332	26.0	74.0	0.375
	Controls	12	37	51		30.5	69.5	
								
	SNP5	C/C	C/G	G/G		C	G	
	Schizophrenics	39	48	13	0.882	63.0	37.0	> 0.999
	Controls	40	45	15		62.5	37.5	
								
	SNP6	C/C	C/G	G/G		C	G	
	Schizophrenics	10	43	47	0.783	31.5	68.5	0.595
	Controls	13	43	44		34.5	65.5	
								

*SLC6A9*	SNP1	C/C	C/T	T/T		C	T	
	Schizophrenics	32	48	20	0.987	56.0	44.0	> 0.999
	Controls	31	49	20		55.5	44.5	
								
	SNP2	A/A	A/G	G/G		A	G	
	Schizophrenics	7	32	61	0.988	23.0	77.0	> 0.999
	Controls	7	31	62		22.5	77.5	
								
	SNP3	A/A	A/G	G/G		A	G	
	Schizophrenics	6	31	63	0.830	21.5	78.5	0.720
	Controls	6	35	59		23.5	76.5	

*Fisher's exact probability tests, case vs. control (2 × 3).

**Fisher's exact probability tests, case vs. control (2 × 2).

### Pairwise linkage disequilibrium and haplotype association analyses

**Figure 2:**
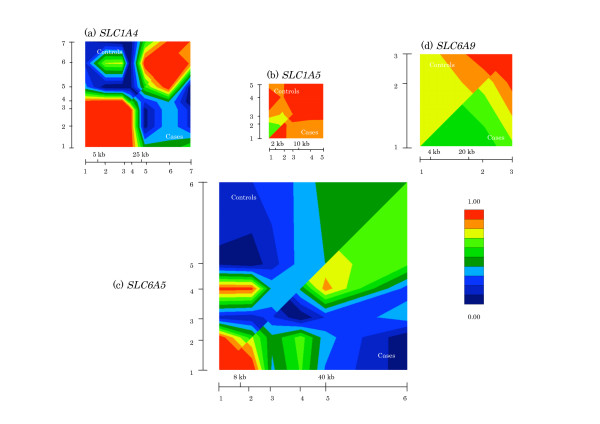
**Pairwise LD analyses using GOLD for control (upper diagonal) and case (lower diagonal) haplotypes of each gene.** The relative location of markers used to construct the haplotypes is represented on the horizontal and vertical axes, which is more clearly depicted in Figure. 1. LD measure, *D'*, is graphically represented adjacent to each GOLD plot (red and dark blue are opposite ends of the scale).

We compared the magnitude of LD for all possible pairs of the SNPs in each gene region in controls and cases by calculating *D' *(Figure 2). No essential difference was shown in the LD pattern of any genes between cases and controls. Strong or modest LD (*D' *> 0.3) were observed in all combinations of adjacent SNPs in *SLC1A5 *and *SLC6A9 *regions. Whereas in each small subregion of the other two gene regions, LD drops abruptly: SNP4-SNP5 of *SLC1A4 *and SNP2-SNP3 of *SLC6A5 *(*D' *= 0.061 and *D' *= 0.125, respectively).

We constructed pairwise haplotypes for all of the possible SNP pairs (Table 2). We observed significant associations with schizophrenia in combinations of SNP2-SNP7 of *SLC1A4 *(*P *= 0.037) and SNP1-SNP4 of *SLC6A5 *(*P *= 0.043). However, neither of them survived after controlling the FDR at level 0.05.

**Table 2:** Association analyses of pairwise haplotypes of SNPs in the genes

		SNP1	SNP2	SNP3	SNP4	SNP5	SNP6
*SLC1A4*	SNP2	0.118					
	SNP3	0.147	0.082				
	SNP4	0.178	0.096	0.132			
	SNP5	0.581	0.114	0.147	0.615		
	SNP6	0.079	0.050	0.062	0.239	0.401	
	SNP7	0.118	0.037	0.057	0.646	0.144	0.377
							
*SLC1A5*	SNP2	0.291					
	SNP3	0.978	0.845				
	SNP4	0.720	0.936	0.999			
	SNP5	0.984	0.944	1.000	0.483		
							
*SLC6A5*	SNP2	0.209					
	SNP3	0.241	0.178				
	SNP4	0.043	0.080	0.424			
	SNP5	0.243	0.160	0.936	0.083		
	SNP6	0.348	0.275	0.932	0.724	0.806	
							
*SLC6A9*	SNP2	0.854					
	SNP3	0.940	0.362				

*P *values by the two-tailed χ^2^-test (d.f. = 3).

### Association analyses using the Full-size Sample Set

Since nominally significant single-marker and haplotype associations with schizophrenia were observed in the Screening Sample Set, we genotyped the Full-size Sample Set for the SNPs involved in the significance, SNP2, SNP3 of *SLC1A4 *and SNP1, SNP2, SNP4 of *SLC6A5 *to examine these significant associations in the Full-size Sample Set. We excluded SNP7 of *SLC1A4 *from further analyses in the Full-size Sample Set because of the insufficient *D' *observed in the combination of SNP2-SNP7 in both cases and controls (*D' *= 0.064 and *D' *= 0.171, respectively). The genotype and allele frequencies of each SNP in the Full-size Sample Set are shown in the Additional File 2. The significant association of SNP1 of *SLC6A5 *with schizophrenia was confirmed in both genotype and allele frequencies in the Full-size Sample Set (*P *= 0.032, *P *= 0.018, respectively). We failed to detect other single-marker associations (*P *value range 0.065 – 0.355) and the haplotype association (*P *= 0.088) observed in the initial screening.

## Discussion

*SLC1A4*, *SLC1A5*, *SLC6A5 *and *SLC6A9 *were located on chromosome 2p13-15, 19q13.3, 11p15.2-p15.1, and 1p33, respectively. Suggestive evidence for linkage of chromosome 2p14-p13, where *SLC1A4 *is located, with schizophrenia has been reported in schizophrenia families from Palau and Ireland [35,36]. However, the subsequent mutation screening failed to find any sequence polymorphism segregated with the illness in the *SLC1A4 *region of the Palauan families [37]. In addition, negative association of *SLC1A4 *with schizophrenia was reported in the German population [38]. There has been no linkage with schizophrenia reported to the chromosome regions where *SLC1A5*, *SLC6A5 *or *SLC6A9 *are located [36]. Moreover, exclusion of linkage between schizophrenia and *SLC1A5 *in 23 English and Icelandic schizophrenia families was reported [39]. Recently, negative associations of schizophrenia with polymorphisms in *SLC6A9 *and *SLC6A5 *were reported in the Chinese and the German population, respectively [40,41]. We investigated the association of *SLC1A4*, *SLC1A5*, *SLC6A5 *and *SLC6A9 *genes with schizophrenia in the Japanese population by analysing total 21 common SNPs.

Since the frequencies of genotyped SNPs are over 0.3, the expected detection powers of the four genes are over 0.80, assuming the genotype relative risk of 1.8. However, assuming lower genotype relative risk of 1.5 or 1.3, the expected detection powers for the four genes dropped to 0.50 – 0.53 or 0.24 – 0.25, respectively. Consequently, the negative finding for genes and SNPs excluded from the analyses using the Full-size Sample Set in this study may be due to type II error at lower relative risks, and they need to be investigated further in an enlarged sample size.

Out of the 21 SNPs analyzed, two within *SLC1A4*, (SNP4 and SNP6, 330 cases and 319 controls) and one within *SLC6A5*, (SNP5, 328 cases and 307 controls) have recently been reported to show no association with schizophrenia in the German population [38,41]. We also observed no association of these SNPs with the disease in our Screening Sample Set. The SNP1 in *SLC6A5 *of which we observed a significant association with the disease, was not included in the report mentioned above.

In LD analysis of the initial screening of the 21 SNPs distributed in the four genes, modest LD (*D' *> 0.3) was observed in all combinations of adjacent SNPs in controls except for the combinations of SNP4-SNP5 of *SLC1A4 *and SNP2-SNP3 of *SLC6A5*, suggesting recombination hot spots in the two regions (6.6 kb and 7.6 kb, respectively) (Figure 2). We compared the LD structure to the publically open database, HapMap [42]. The LD gap we observed in the *SLC6A5 *region was not observed in the HapMap LD structure from either Japanese or Chinese population data (*D' *= 0.817 and *D' *= 1, respectively). The other LD gap, which was observed in the *SLC1A4 *region, failed to be compared due to the absence of the novel SNP we found.

We observed significant single-marker associations in SNP2 and SNP3 of *SLC1A4 *in the Screening Sample Set. However, we failed to confirm these findings in the Full-size Sample Set. We attribute to type I error due to the small sample size used in the Screening Sample Set. On the other hand, the single-marker association of SNP1 (rs894747) in *SLC6A5 *region, although it does not show the significant association with the disease in the independent 300 case and 320 control samples (0.092), it does show the significant association in the Full-size Sample Set (*P *= 0.018). We consider that the nonsignificant result observed in the enlarged samples may be due to the small sample size. SNP1 is located in the intergenic region, 2,355-bp upstream from the transcription start site. In the negative association report of *SLC6A5 *in German population described above, four SNPs and one short-tandem-repeat distributed in intron 1~intron 11, but no SNP located in the upstream region were analysed [39]. In our Full-size Sample Set, the G allele was more frequently observed in schizophrenics (44.4%) than in controls (38.6%). Therefore, the G allele may be in LD with a risk allele for schizophrenia (odds ratio, 1.27; 95% confidence interval, 1.04~1.55). We conclude that at least one susceptibility locus for schizophrenia is located within or nearby *SLC6A5*, whereas *SLC1A4 SLC1A5 *and *SLC6A9 *are unlikely to be major susceptibility genes for schizophrenia in the Japanese population. No potential regulatory elements were previously identified in the region where SNP1 is located [43]. It is necessary to search for functional SNPs in the haplotype block where SNP1 is located. A copy number variation (CNV) has been reported in the European population on the chromosome 11p15.1, containing exon 15 of *SLC6A5 *[44]. None of the 6 SNPs we genotyped is located within the CNV. Although the frequency of the CNV in the Japanese population is unknown, SNP1 may be associated with the variant devoid of exon 15, which is a strong candidate of the susceptible allele. Therefore, it is necessary to test the association of the CNV with schizophenia in Japanese sample sets. The positive association observed in *SLC6A5 *also needs to be validated in different ethnic populations.

## Conclusion

We conclude that at least one susceptibility locus for schizophrenia is located within or nearby *SLC6A5*, whereas *SLC1A4 SLC1A5 *and *SLC6A9 *are unlikely to be major susceptibility genes for schizophrenia in the Japanese population.

## Competing interests

The authors declare that they have no competing interests.

## Authors' contributions

XD carried out a portion of genotyping, statistical analyses and drafted the manuscript; NS, NT, MT carried out a portion of genotyping and statistical analyses; HN, NI and NO participated in collecting specimens and clinical data; HS participated in design of this study and statistical analyses; YF conceived of the study and participated in its design and coordination. All authors read and approved the final manuscript.

## Pre-publication history

The pre-publication history for this paper can be accessed here:



## Supplementary Material

Additional file 1 PCR primers for genotyping of SNPs in the genes. The data provided the nucleotide sequences of primers, PCR conditions and genotyping methods for each SNP. Click here for file

Additional file 2 Genotype and allele frequencies of SNPs in the Full-size Sample Set (400 cases and 420 controls). The data show genotype and allele frequencies of SNPs in the Full-size Sample Set. Click here for file
